# Experimental and numerical simulation dataset of a ferrocement wall subjected to fully-reversed cyclic load test

**DOI:** 10.1016/j.dib.2024.110095

**Published:** 2024-01-24

**Authors:** Bryan Chalarca, Giammaria Gabbianelli, Daniel Bedoya-Ruiz, Roberto Nascimbene

**Affiliations:** a Department of Civil Engineering and Architecture, University of Pavia, Pavia, Italy; b Universidad Nacional de Colombia – Sede Manizales, Manizales, Colombia; c University School for Advanced Studies IUSS Pavia, Pavia, Italy

**Keywords:** Hysteretic response, Nonlinear model, Low-cost building materials, In-plane response

## Abstract

The need for affordable housing in developing countries requires the development and use of new structural systems that allow easy, fast, and cheap construction while providing a sufficient seismic performance to protect the life and belongings of the population. In this regard, structural wall systems based on ferrocement have proved to be a feasible option to meet these criteria. Ferrocement is a construction system composed of mortar (i.e., cement, sand, and water) and thin and closely spaced steel reinforcement (i.e., small rebars, welded mesh, hexagonal wire mesh, metal fibers, etc.). This dataset corresponds to the hysteretic response of a ferrocement wall subjected to an increasing in-plane fully-reversed cyclic load test, following the loading protocol given by the ASTM Standard E2126-11. The dataset is composed of the forces and displacements read at the top section of the wall specimen. Finally, a nonlinear finite element model of the ferrocement wall, developed using SeismoStruct software, is also included along with the equivalent numerical simulation of the experimental test. This dataset can be potentially used for the calibration of hysteretic models with high pinching levels and the structural identification of ferrocement walls.

Specifications Table

**Table:** 

Subject	Civil and Structural Engineering
Specific subject area	Fully-reversed cyclic test, hysteretic response, and numerical simulation of a ferrocement wall specimen with a cement-woodchip core.
Data format	Postprocessed displacement (mm) and force (kN) data obtained from the experimental test and the equivalent numerical simulation on SeismoStruct software on .csv files.
Type of data	.csv files (dataset with numbers). .spf file (numerical model on SeismoStruct software). .xlm file (equivalent numerical of the SeismoStruct file). .pdf file (test setup and wall specimen geometry). .txt (general information). .jpg (specimen pictures).
Data collection	The ferrocement wall specimen was embedded in a reinforced concrete foundation beam, which in turn was fixed to the reaction floor of the structural laboratory through 25.4 mm (1-inch) steel stud bolts to restrain relative displacements between the wall specimen and its foundation. Out-of-plane constraints were implemented using lateral bracing to avoid possible eccentricities in the in-plane movement generated by a hydraulic actuator attached to the top of the specimen through a loading concrete beam. The forces were recorded by a load cell at the head of the hydraulic actuator and the displacements by a linear variable differential transformer (LVDT) placed between the hydraulic actuator's head and its body.
Data source location	The experimental campaign was conducted at the Structures Laboratory of the Universidad Nacional de Colombia – Sede Manizales, in Manizales, Colombia.
Data accessibility	Repository name: Open Science Framework Data identification number: 10.17605/OSF.IO/Z2M7A Direct URL to data: https://osf.io/z2m7a/

## 1 Value of the Data

• This dataset set is valuable due to the expenses, infrastructures, and equipment required to conduct fully-reversed cyclic load tests on full-size wall specimens. The hysteretic response obtained from this test is crucial for the structural identification of the tested ferrocement wall.
• This dataset can be used as a research reference for homologation processes by building code agencies of structural systems based on ferrocement systems.
• The experimental hysteric response values contained in the dataset can be used for the development and validation of hysteretic models characterised by strong pinched hysteresis loops. In addition, the provided images of the final state of the wall specimen can help understand the induced damage in this type of structural configuration.
• The proposed nonlinear numerical model can serve as a reference point for further evaluation of the tested structural system, especially considering software limitations to model thin ferrocement walls.

## 2 Background

The threat posed by middle and strong earthquakes remains a considerable hazard for communities globally. Over the last two decades (2000-2019), these seismic events accounted for 58% of the total number of deaths resulting from natural disasters. This is particularly pronounced in countries with deficient building codes and limited construction quality, as highlighted by the United Nations Office for Disaster Risk Reduction [1]. In addition, the increasing urban population has generated a large demand for affordable housing, creating a significant need to propose structural systems that allow safe, cheap, fast, and easy construction, aiming to fulfil the objectives of the UN Sendai Framework for Disaster Risk Reduction 2015-2030 [2]. In this regard, the structural properties and easy implementation of ferrocement systems can represent a feasible solution for the development of affordable housing in developing countries. The experimental and numerical results presented herein help achieve this objective by providing crucial data for further research and development of structural systems with the aforementioned qualities.

## 3 Data Description

**Fig. 1:**
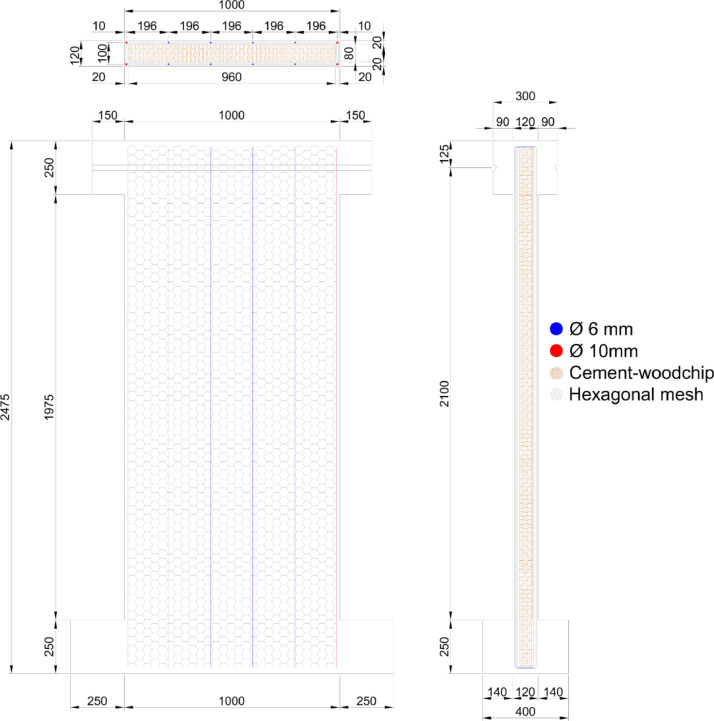
Geometry of the ferrocement wall specimen.

**Fig. 2:**
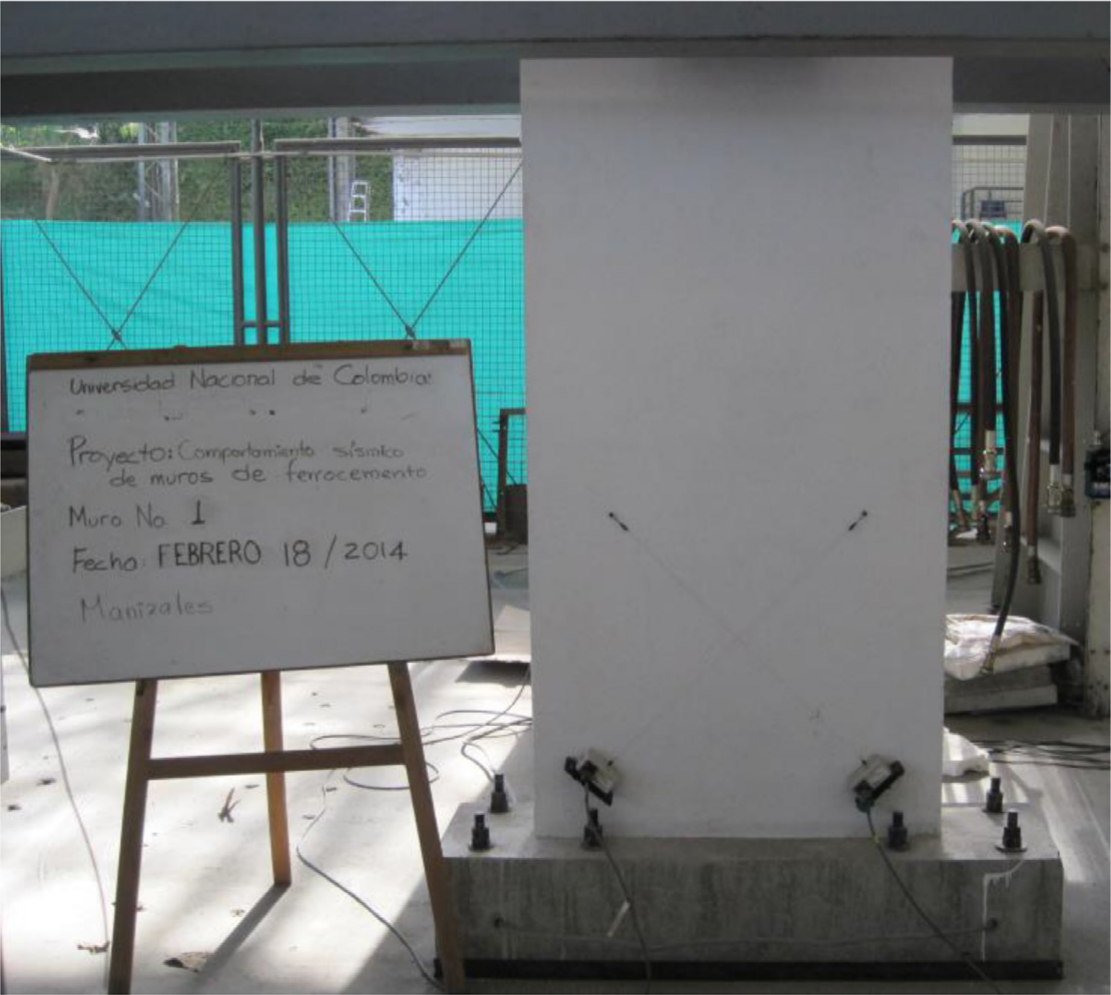
Ferrocement wall specimen.

**Fig. 3:**
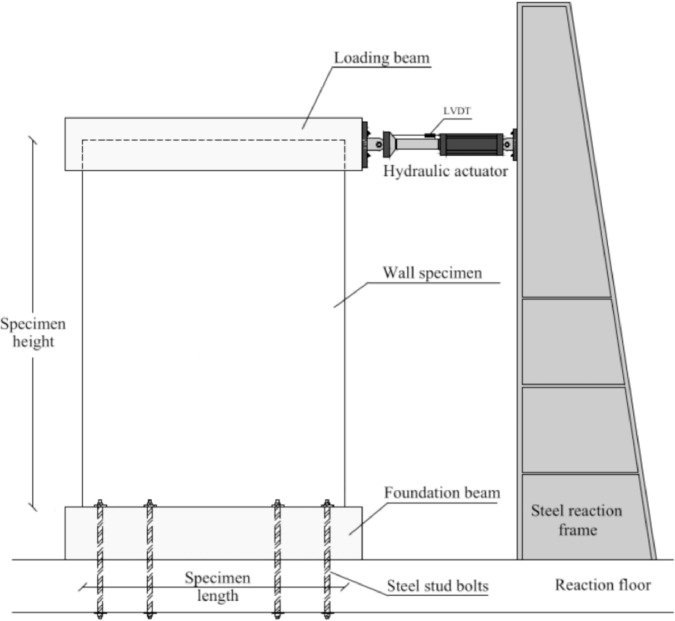
Scheme of the test setup.

**Fig. 4:**
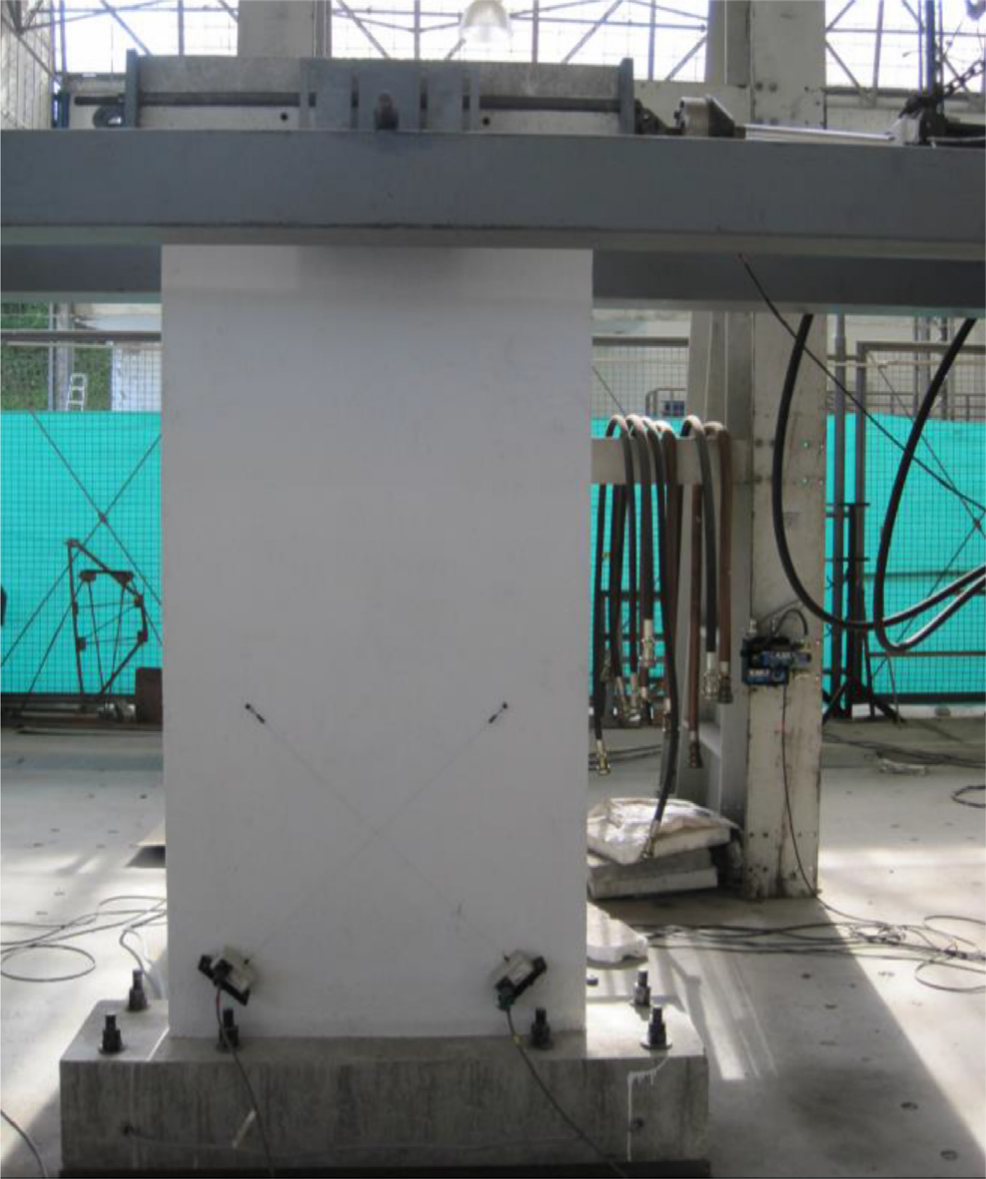
Test setup front view.

**Fig. 5:**
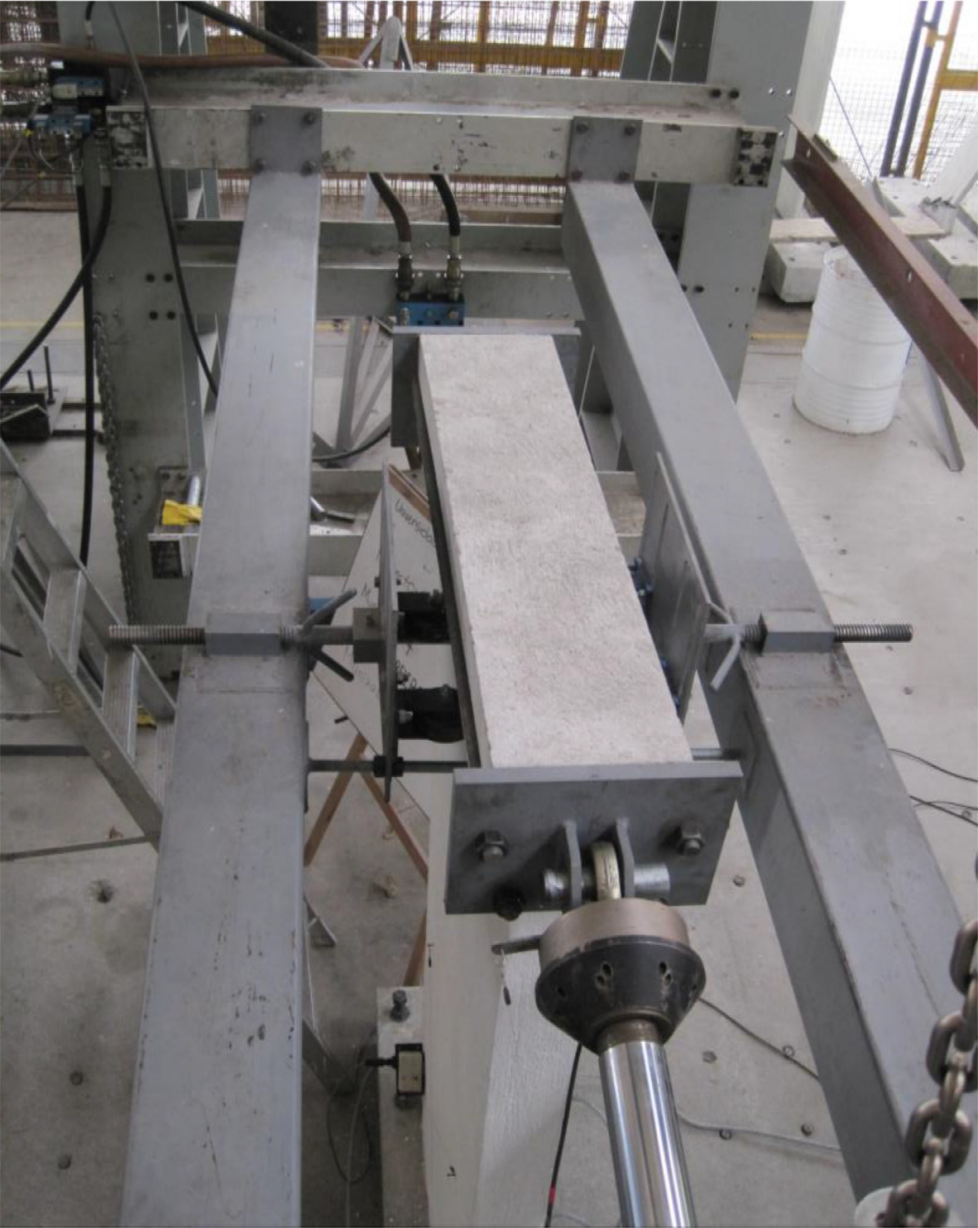
Test setup top view.

**Fig. 6:**
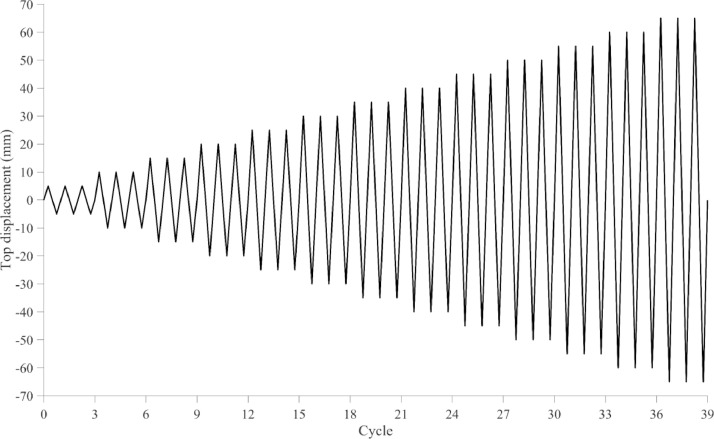
fully-reversed cyclic load test based on the ASTM Standard E2126-11 [4].

**Fig. 7:**
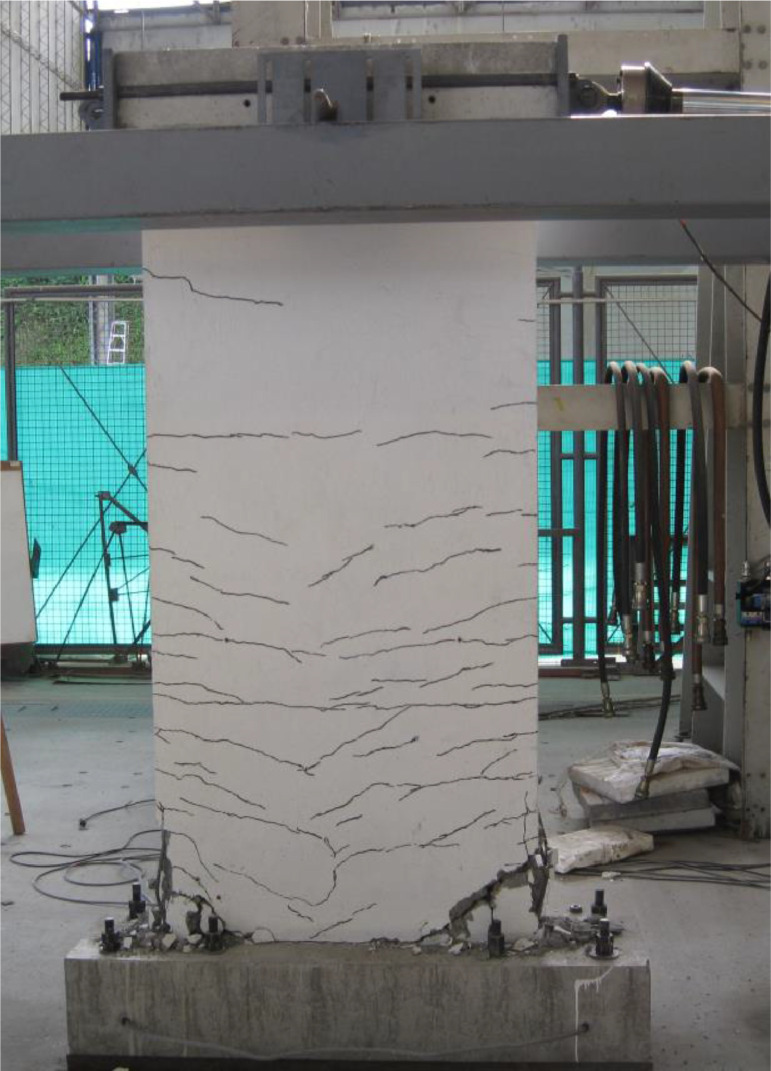
Final state of the ferrocement wall specimen, front view.

**Fig. 8:**
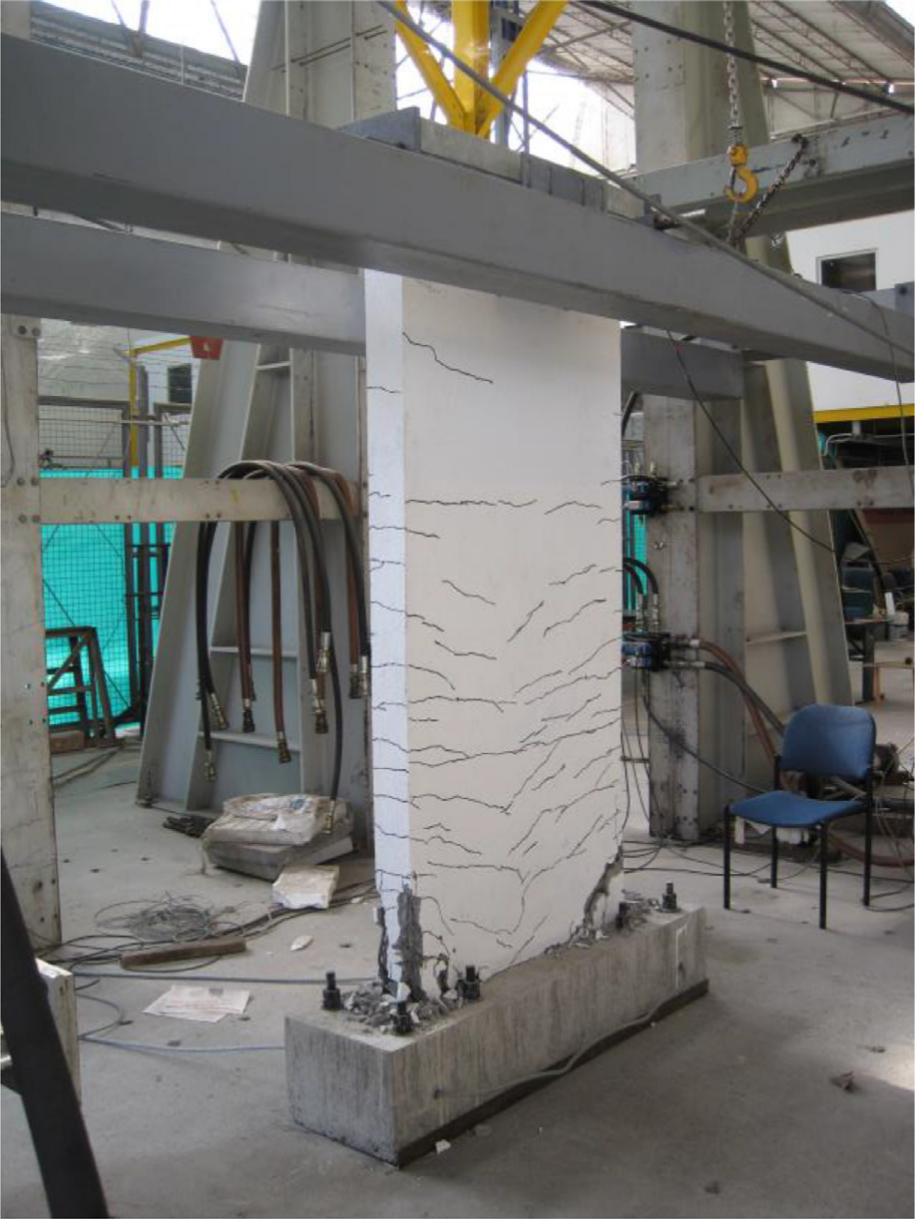
Final state of the ferrocement wall specimen, isometric view.

**Fig. 9:**
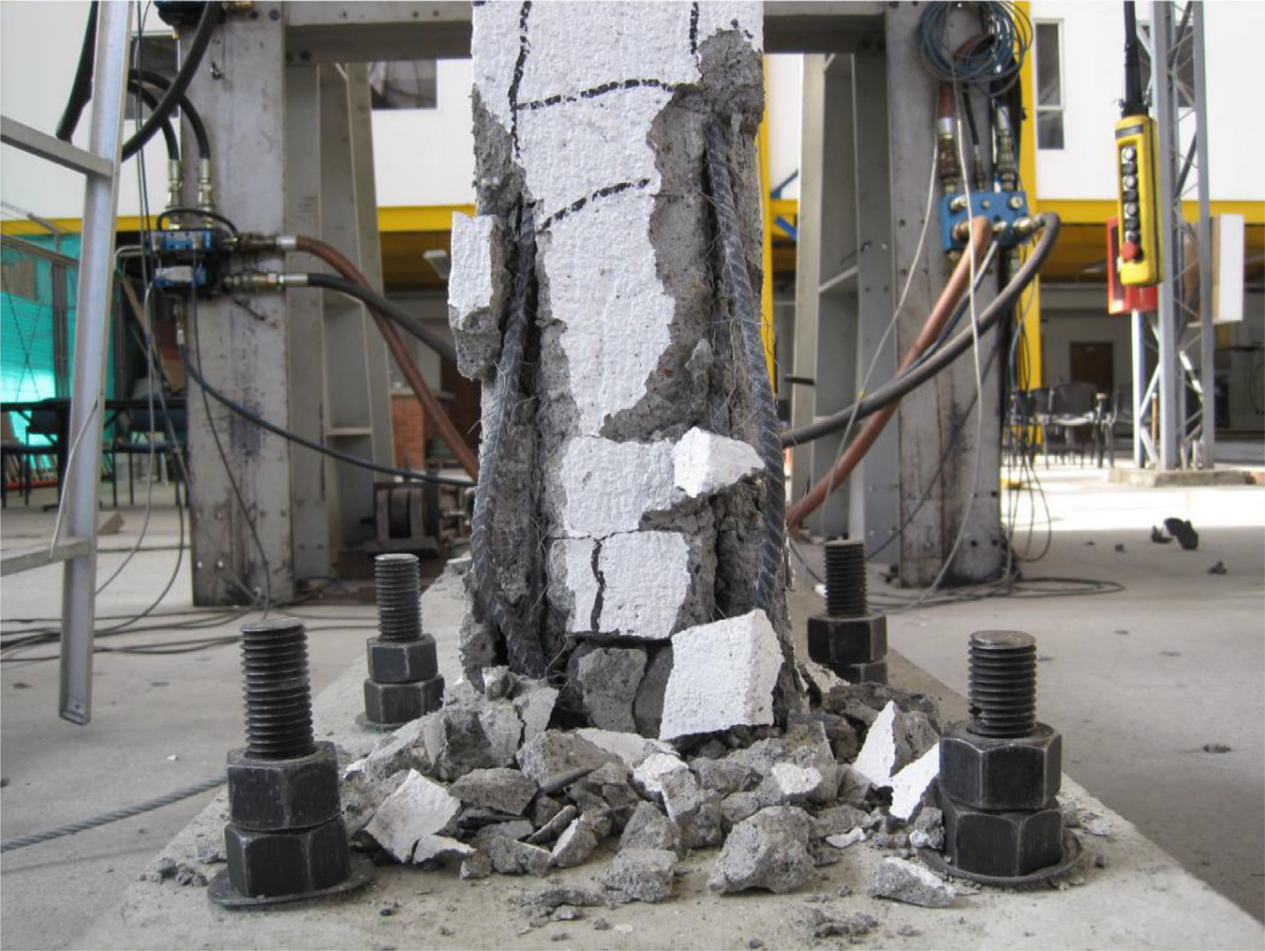
Specimen wall toe damage, left close view.

**Fig. 10:**
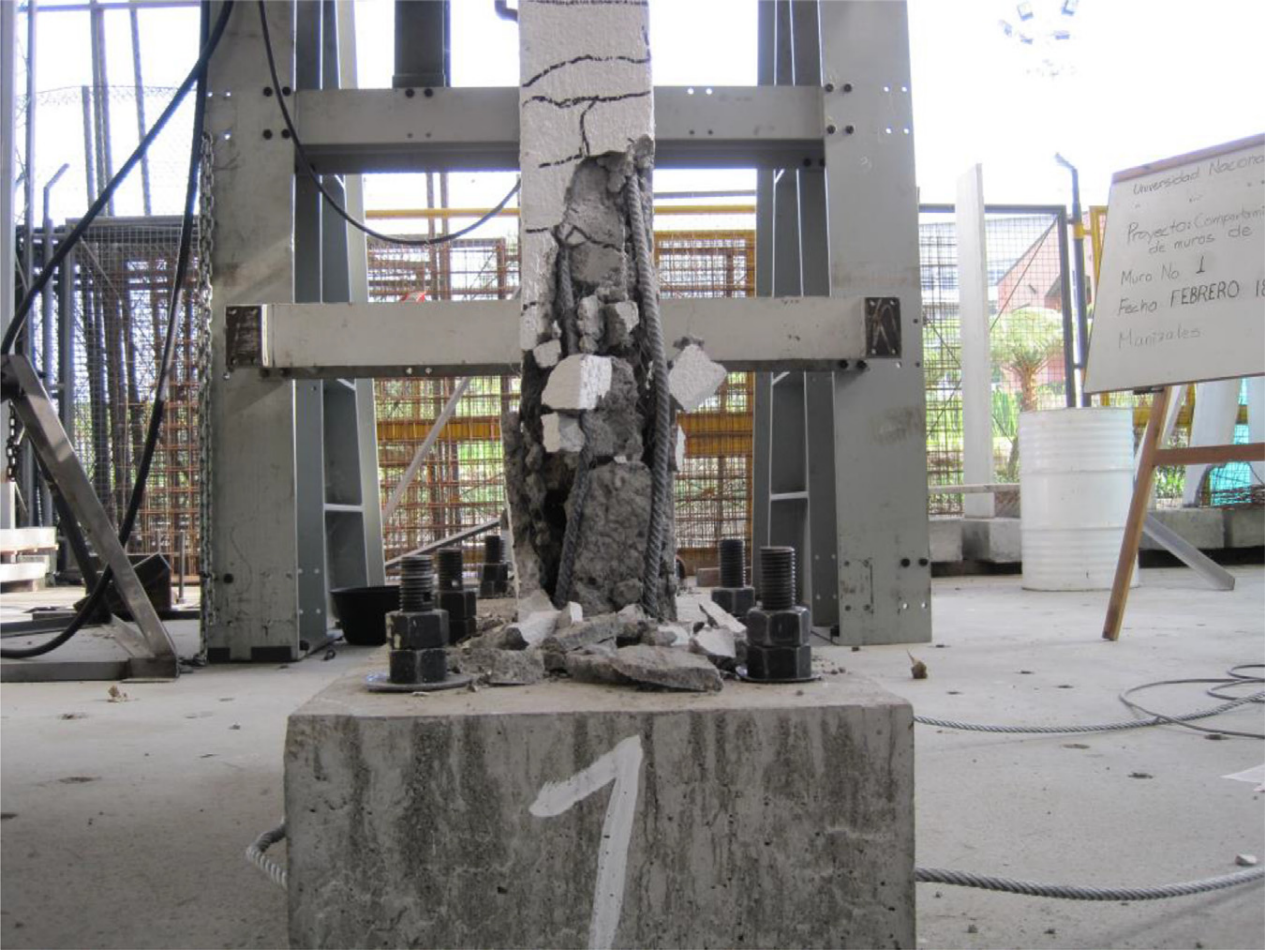
Specimen wall toe damage, right close view.

**Fig. 11:**
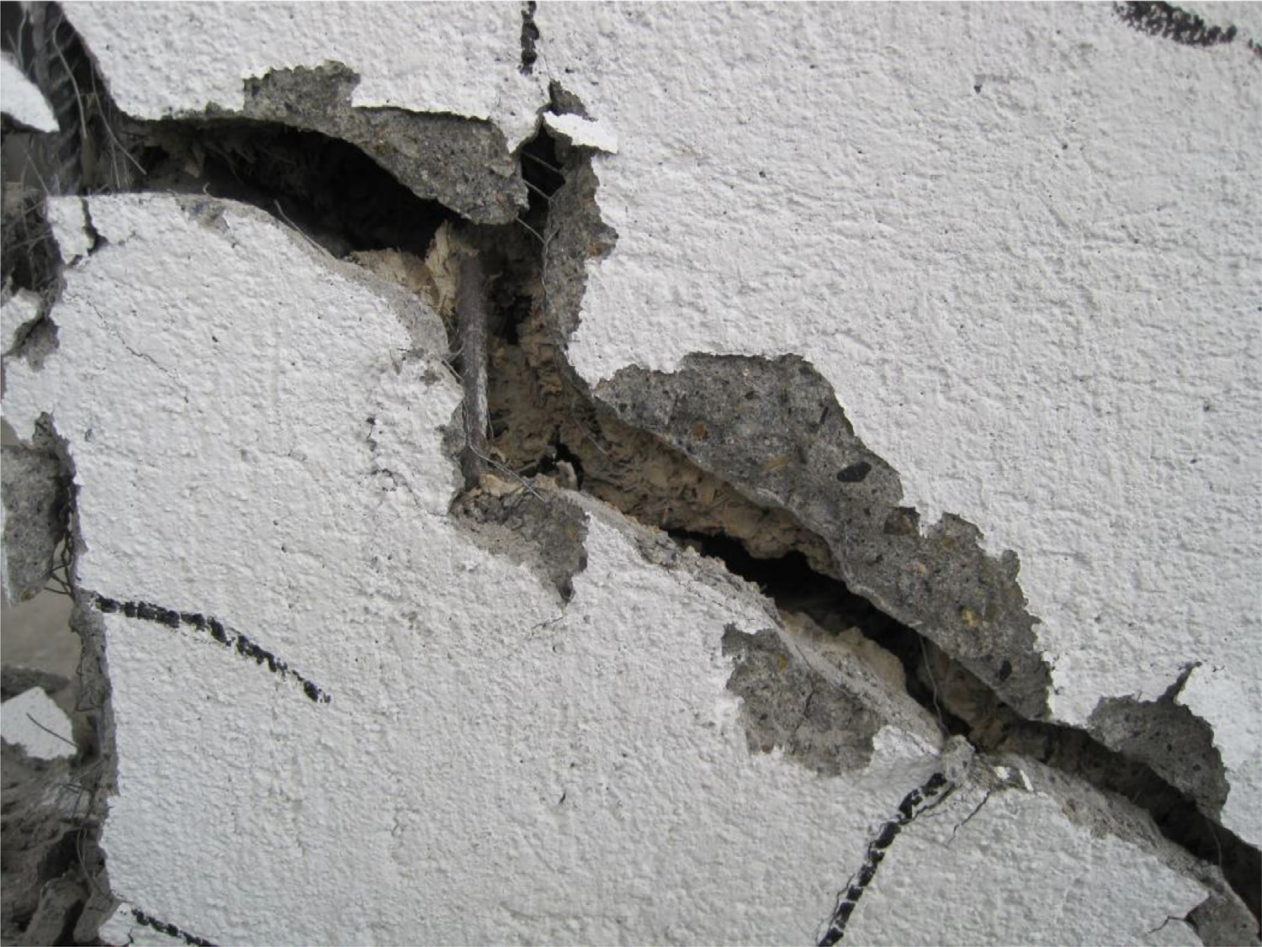
Cement-woodchip core damage, close view.

The dataset “Hysteretic Response of Ferrocement Walls” [3] comprises three folders and a readme file with general comments on the available data. The first folder, “Dataset – Hysteretic Response”, contains two .csv files that correspond to the experimental hysteretic response (test_data_mm_kN.csv) and numerical hysteretic response (numerical_data_mm_kN.csv). Each .csv file is composed of two columns containing the displacement in mm and the force in kN, respectively, of the hysteretic response of the ferrocement wall. Finally, a figure (Hysteretic response.tif) shows both the experimental and the numerical hysteretic response of the ferrocement wall specimen. The second folder, “Images”, contains two figures, showing the experimental setup (Fig. 1 Test setup.pdf) and the ferrocement wall geometry (Fig. 2 Wall geometry.pdf), and nine pictures of the actual ferrocement wall specimen, showing the wall before (Fig. 3, Fig. 4) and after (Fig. 5, Fig. 6, Fig. 7, Fig. 8, Fig. 9, Fig. 10, Fig. 11) the fully-reversed cyclic load test. Finally, the third folder, “Numerical model”, contains the nonlinear numerical model (Ferrocement_wall_cyclic.spf), the equivalent human-readable numerical model (Ferrocement_wall_cyclic.xml), and the input load pattern (cyclic.txt) used to simulate the results obtained from the experimental test. The nonlinear numerical model was generated on the SeismoStruct software v2023 Released-2 Built-50 using an academic license.

## 4 Experimental Design, Materials and Methods

### 4.1 Description of the ferrocement wall

The ferrocement wall specimen (see Fig. 1) had a cross-section dimension of 1000 mm by 120 mm and was composed of a 20 mm layer of ferrocement covering a cement-woodchip core. The cement-woodchip core had a compressive strength of 5 MPa with a cross-section dimension of 960 mm by 80 mm. The ferrocement reinforcement was composed of four rebars with a diameter of 10 mm at the corners of the specimen and eight rebars with a diameter of 6 mm distributed along both sides of the specimen. In addition, eight layers of hexagonal mesh with a diameter of 1 mm and an opening equal to 31.75 mm surrounded the rebars from the interior (four layers) and exterior (four layers) sides. The rebars and the hexagonal mesh were characterised by a yield strength of 420 MPa and 282 MPa, respectively. Finally, the mortar was composed of Portland cement type I, sand without coarse aggregate, water, and a superplasticiser additive to improve the manageability and penetrability of the mortar through the reinforcement. The mortar had a compressive strength of 44 MPa after 28 days. Fig. 2 shows the ferrocement wall specimen. The construction process of the ferrocement wall specimen started with casting the cement-woodchip core. Once the core was hard enough to support its weight, it was covered with four layers of hexagonal mesh. Thereafter, the steel reinforcement of the foundation beam was set along with the stud bolt holes and the cement-woodchip core was placed in the centre of the beam. The vertical rebars were added around the cement-woodchip core and were covered with four layers of hexagonal mesh. The cement-woodchip core along with the hexagonal mesh and the vertical rebars were rendered with mortar until reaching the selected thickness. Finally, the foundation beam was cast, and the loading beam was set and cast on top of the ferrocement wall.

## 5 Test Setup

The ferrocement wall specimen was embedded in a reinforced concrete foundation beam, which in turn was fixed to the reaction floor of the structural laboratory through 25.4 mm (1-inch) steel stud bolts to restrain relative displacements between the wall specimen and its foundation. Out-of-plain constraints were implemented on both sides of the wall specimen using lateral rollers to avoid possible eccentricities in the in-plane movement. The induced displacements were generated by a hydraulic actuator, with a maximum capacity of 250 kN, anchored to the steel reaction frame and attached to the top of the wall specimen through the loading concrete beam. The induced displacements were applied with a constant velocity of 0.33 mm/s and were measured by a linear variable differential transformer (LVDT) located between the hydraulic actuator's head and its body. On the other hand, the resulting forces were recorded by a load cell at the head of the hydraulic actuator. The test setup is illustrated in Fig. 3, Fig. 4, Fig. 5. The foundation and loading beam were built using concrete of compressive strength of 28 MPa after 28 days, and their reinforcement was composed of longitudinal rebars with a diameter of 16 mm and stirrups with a diameter of 10 mm every 100 mm. The ferrocement wall specimen was subjected to the fully-reversed cyclic load test protocol proposed by the ASTM Standard E2126-11 [4], which consists of a series of three fully-reversed cycles for each target top displacement, with increments of 5 mm until reaching a target top displacement of 65 mm, attaining a significant reduction of the in-plane capacity of the ferrocement wall specimen. Fig. 6 illustrates the input motion used for the experiment and Fig. 7–11 show the final state of the ferrocement wall specimen after the test.

## 6 Nonlinear Numerical Model

**Fig. 12:**
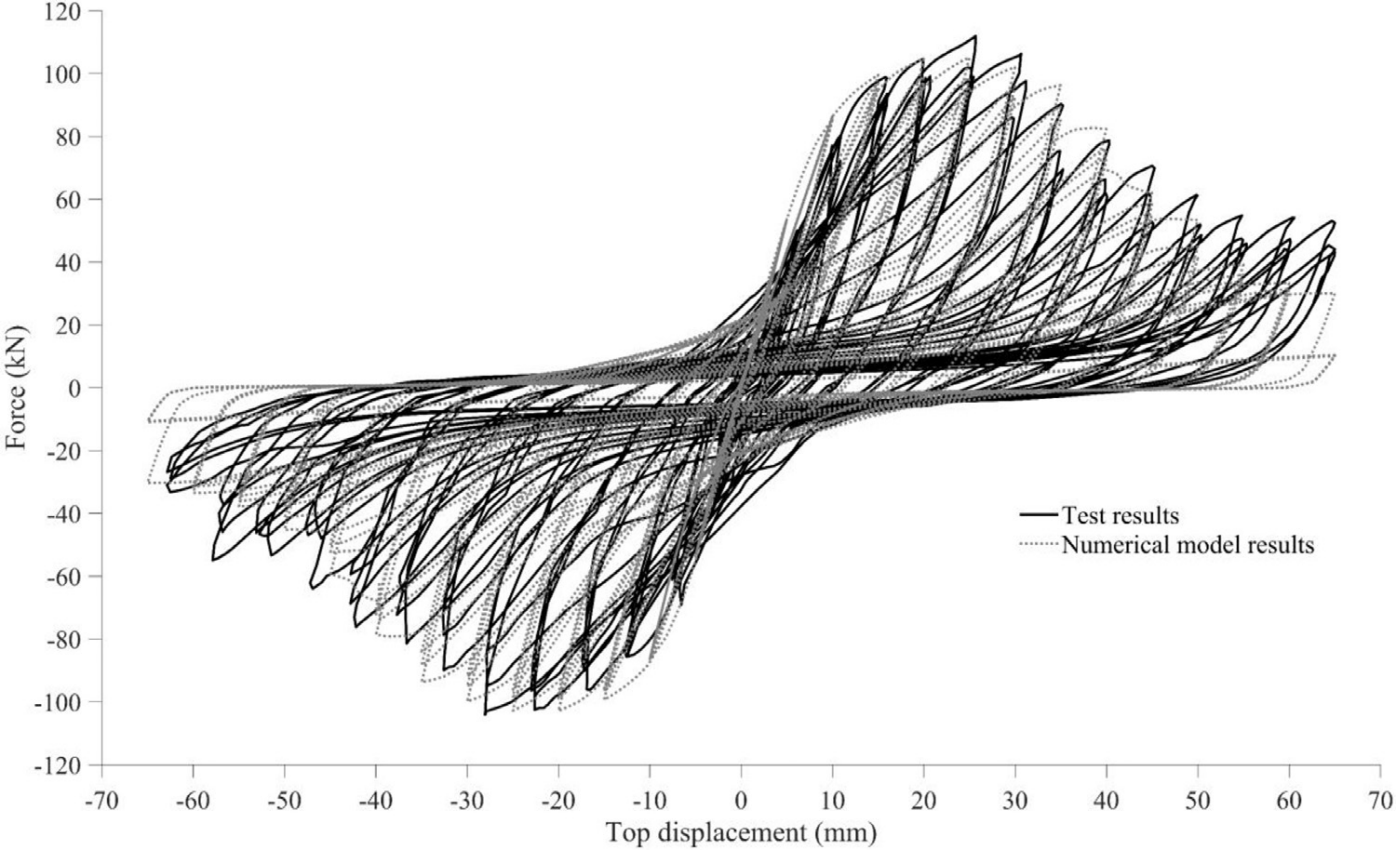
Experimental and numerical hysteretic responses.

The ferrocement wall specimen was modelled using the SeismoStruct software v2023 Released-2 Built-50 using a reinforced rectangular concrete hollow section. The model is composed of three materials that define horizontal reinforcement, vertical reinforcement, and mortar. The horizontal reinforcement was simulated by stirrups equivalent to the horizontal component of the hexagonal mesh layers and characterised by an S220 steel material [5,6]. The material properties are shown in Table 1. The vertical reinforcement was characterised by a hysteric material calibrated to represent the lack of proper confinement that led to the buckling of the vertical rebars, especially those close to the ends of the wall specimen. The mortar was also characterised by a hysteretic material to include the tension capacity provided by the vertical component of the hexagonal mesh layers and the additional compression strength given by the cement-woodchip core. Table 2 reports the material properties of both hysteretic materials. More information on the material properties can be found in the SeismoStruct manual [7]. Finally, Fig. 12 shows the experimental and numerical hysteretic response of the ferrocement wall specimen.

**Table 1:** Material properties of the horizontal reinforcement – steel material.

Property	Value
Modulus of elasticity (GPa)	200.0
Yield strength (MPa)	253.0
Strain hardening parameter	0.005
Transition curve initial shape parameter	20.0
Transition curve shape calibrating coeff. A1	18.5
Transition curve shape calibrating coeff. A2	0.15
Isotropic hardening calibrating coeff. A3	0.00
Isotropic hardening calibrating coeff. A4	1.00
Fracture/buckling strain	0.20

**Table 2:** Material properties of the mortar and vertical reinforcement – hysteretic material.

Property	Mortar value	Vertical rebar value
Modulus of elasticity (GPa)	4.7	200
Yield strength in positive direction (MPa)	7.5	420
Yield strength in negative direction (MPa)	50.0	150
Peak strain in positive direction (m/m)	0.30	0.20
Peak stress in positive direction (MPa)	7.5	483
Peak strain in negative direction (m/m)	0.003	0.20
Peak stress in negative direction (MPa)	50.0	172
Residual strength in positive direction (MPa)	1.0	1.0
Residual strength in negative direction (MPa)	7.5	1.0
Pinching factor	0.25	0.01
Deterioration factor	0.30	0.25

## Limitations

The dataset and other results presented in this paper can be influenced by considering other factors during the experimental campaign, such as out-of-plane or larger vertical loads. In addition, more steel configurations should be tested to provide a more generalized seismic response of ferrocement walls. Another factor that should be further verified is the influence of the cement-woodchip on the final hysteretic response. Finally, a shake-table test should be implemented for more accurate identification of seismic-induced damage on the ferrocement system.

## Ethics Statement

The authors state that the current work does not involve human subjects, animal experiments, or any data collected from social media platforms.

## CRediT authorship contribution statement

**Bryan Chalarca:** Conceptualization, Methodology, Software, Validation, Investigation, Data curation, Writing – original draft, Writing – review & editing. **Giammaria Gabbianelli:** Conceptualization, Methodology, Software, Writing – review & editing. **Daniel Bedoya-Ruiz:** Conceptualization, Validation, Investigation, Resources, Writing – review & editing. **Roberto Nascimbene:** Conceptualization, Writing – review & editing.

## Data Availability

Hysteretic Response of Ferrocement Walls (Original data) (Open Science Framework)
